# Environmentally induced variation in sperm sRNAs is linked to gene expression and transposable elements in zebrafish offspring

**DOI:** 10.1038/s41437-025-00752-2

**Published:** 2025-03-22

**Authors:** Alice M. Godden, Willian T. A. F. Silva, Berrit Kiehl, Cécile Jolly, Leighton Folkes, Ghazal Alavioon, Simone Immler

**Affiliations:** 1 School of Biological Sciences, University of East Anglia, Norwich Research Park, Norwich, NR4 7TJ UK; 2 Uppsala University, Department of Evolutionary Biology, Norbyvägen 18D, 75310 Uppsala, Sweden; 3 Department of Physics, Chemistry and Biology, Linköping University, 58183 Linköping, Sweden

**Keywords:** Evolutionary genetics, Bioinformatics

## Abstract

Environmental factors affect not only paternal condition but may translate into the following generations where sperm-mediated small RNAs (sRNAs) can contribute to the transmission of paternal effects. sRNAs play a key role in the male germ line in genome maintenance and repair, and particularly in response to environmental stress and the resulting increase in transposable element (TE) activity. Here, we investigated how the social environment (high competition, low competition) of male zebrafish *Danio rerio* affects sRNAs in sperm and how these are linked to gene expression and TE activity in their offspring. In a first experiment, we collected sperm samples after exposing males to each social environment for 2 weeks to test for differentially expressed sperm micro- (miRNA) and piwi-interacting RNAs (piRNA). In a separate experiment, we performed in vitro fertilisations after one 2-week period using a split-clutch design to control for maternal effects and collected embryos at 24 h to test for differentially expressed genes and TEs. We developed new computational prediction tools to link sperm sRNAs with differentially expressed TEs and genes in the embryos. Our results support the idea that the molecular stress response in the male germ line has significant down-stream effects on the molecular pathways, and we provide a direct link between sRNAs, TEs and gene expression.

## Introduction

The importance of non-genetic inheritance of environmentally induced variation in paternal condition for offspring fitness is increasingly accepted (Bonduriansky et al. 2012; Bonduriansky and Day 2009). A range of factors affect not only the condition of the father but also the resulting offspring including temperature (Salinas et al. 2013; Salinas and Munch 2012), diet (Dunn and Bale 2009; Huypens et al. 2016; Valtonen et al. 2012), social interactions (Saavedra-Rodriguez and Feig 2013), and stress (Franklin et al. 2010; Kan et al. 2016; Weiss et al. 2011; Zaidan et al. 2013). While it is generally accepted that these effects may be transferred through epigenetic (namely, non-DNA encoded) factors such as methylation patterns (Jiang et al. 2013; Potok et al. 2013), chromatin structure (Lindeman et al. 2011) and small RNAs (sRNAs) (Gapp et al. 2014; Houri-Zeevi et al. 2020; Rechavi et al. 2014), it is unclear whether these factors ending up in sperm are adaptive or part of the repair and maintenance response of the male germ line to environmental stress (Godden and Immler 2023). Here we focused on the possible role of sperm sRNAs in the transmission of inter-generational paternal effects and their link to transposable element (TE) activity and gene expression in the resulting offspring.

Among the sperm-mediated sRNAs, micro- (mi-) and piwi-interacting (pi-) RNAs are known to exhibit differential expression in sperm and may contribute to non-genetic inheritance (Immler 2018; Rando 2016). In adult male Norway rats *Rattus norvegicus* for example, heat stress altered gene expression as well as miRNA expression in spermatocytes and spermatids (Yadav et al. 2018), and in zebrafish *Danio rerio*, thermal stress affected the miRNA profile in testes (van Gelderen et al. 2022). MiRNA and piRNA profiles in sperm of male house mice *Mus musculus* exposed to trauma as juveniles differed from the profiles in sperm of control mice, and these differences in sRNA expression were directly linked to changes in the behaviour of the resulting offspring (Gapp et al. 2020). Finally, long-term exposure to different sex ratios and diets in selection lines of the fruit fly *Drosophila melanogaster* led to differences in sperm miRNA profiles between the different lines (Hotzy et al. 2021).

However, sRNAs also have a key role in gene regulation and DNA maintenance and repair in the animal germ line (Bartel 2004; Godden and Immler 2023; Houwing et al. 2007). MiRNAs post-transcriptionally regulate gene expression through complementary seed region binding of the mature miRNA and the 3’ untranslated region of a mRNA; one miRNA can regulate many hundreds of genes, and as a result can fine-tune gene expression (Agarwal et al. 2015; Bartel 2004; Thatcher et al. 2008). PiRNAs are involved in protecting the genome, particularly the germline genome from invasive TEs (Chang et al. 2022; Houwing et al. 2007). DNA transposons are the most abundant TEs in the zebrafish genome and contribute to the epigenetic state of the genome (Chang et al. 2022). Many DNA transposons exhibit testes-specific expression in zebrafish suggesting they may have tissue-specific regulatory functions (Lee et al. 2022). Long Terminal Repeat (LTR) elements are enriched very early in zebrafish embryo development during post-zygotic genome activation, and DNA TEs are expressed in later development (Chang et al. 2022). The enrichment for piRNAs complementary to LTR elements at these early stages of embryo development may therefore offer protection to the developing zebrafish germline in changing environments (Chang et al. 2022; Houwing et al. 2007).

While environmental factors such as temperature, diet and toxins are directly interacting with physiological processes (Barrionuevo and Burggren 1999; Ton et al. 2003), the effects of the social environment on individuals is more complex. A common response to competitive social interactions is stress, which can potentially cause diseases in the individuals directly exposed to it (Garratt et al. 2016; McEwen and Stellar 1993) and can affect the next generation (Franklin et al. 2010; Kan et al. 2016; Weiss et al. 2011; Zaidan et al. 2013). In fact, stressful environments lead to higher cortisol levels and in extreme cases death in subordinate zebrafish, as zebrafish act aggressively and will establish dominance if kept in pairs (Dahlbom et al. 2011). As a consequence, social interactions and social hierarchy among males during competition for access to females are known to affect ejaculate and sperm traits in a wide variety of species (Fitzpatrick and Lupold 2014), and dominant males produce more sperm compared to subordinate males (Filby et al. 2010). Stress experienced by males may also carry over into the following generations, potentially through the transmission of sRNAs in sperm. In male zebrafish for example, stress induced by an alarm cue prior to mating led to differential expression in sRNA profiles in sperm, where 12 out of 213 mature miRNAs, 6 out of a 569 piRNA clusters (all enriched), and 12 out of 55 tsRNA clusters showed differential expression (Ord et al. 2020). In addition, offspring of these stressed males exhibited altered stress responses, and increased thigmotaxis. We previously showed that the intensity of male-male competition experienced by fathers prior to siring offspring affects offspring performance, with males under high competition siring faster hatching offspring with lower survival rates (Zajitschek et al. 2014). Similarly, the social status of zebrafish males immediately before fertilisation affected offspring activity, and changes in social hierarchy had the strongest effects where males shifting from dominant to subordinate seemed to be experiencing the biggest change (Zajitschek et al. 2017). The mechanisms of these observed effects on offspring phenotype are currently not known.

The aim of our study was to understand how variation in the paternal social environment may affect sRNA profiles in sperm and whether these are linked to gene expression and TE activity during early embryo development in the resulting offspring. We exposed male zebrafish to an experimental setup where males were either kept in company of one male and one female (high competition) or in the company of two females (low competition). In a first experiment, we exposed each male to both treatments in random order for 2 weeks each and collected a total of two sperm samples, one at the end of each period. We conducted sRNA sequencing with a focus on mi- and piRNAs on the sperm samples and mRNA sequencing on the embryos to identify differentially expressed genes and TEs. In a second experiment, we exposed males to one of the two treatments for 2 weeks only, and performed in vitro fertilisations (IVF) at the end using a split-clutch design to control for maternal effects. We collected embryos at 24 h post-fertilisation, a key stage in early embryo development with highly synchronised expression patterns (Mathavan et al. 2005), for whole transcriptome analyses. Subsequently, we were able to link differentially expressed sRNAs in sperm with differential expression of putative target genes and TEs in the offspring using newly developed bioinformatic tools.

## Materials and methods

All fish used in the experiment were adult male and female zebrafish from the AB wild-type strain obtained from ZIRC and bred under a carefully designed outbreeding regime for one generation in the SciLifeLab zebrafish facility at Uppsala University. The fish were raised to sexual maturity and kept under standard laboratory conditions at a temperature of 28 °C and a 12:12 h light-dark cycle. The fish were fed ad libitum three times per day with live *Artemia* (ZM Systems, UK) and dry food (Medium granular, ZM Systems, UK). The Swedish ethical standards were followed, and all experimental protocols were approved by the Swedish Board of Agriculture (Jordbruksverket, approval no. C 3/15).

### Experimental set up for sperm sample collection

To collect sperm samples for sRNA-sequencing, males were exposed to one of two different treatments as described above and in (Zajitschek et al. 2014): (1) a high risk of sperm competition treatment (hereafter referred to as High) where two males were kept with one female, or (2) a low risk of sperm competition treatment (Low) where one male was kept with two females in 3 L tanks. In zebrafish, both males and females compete for spawning and both sexes can be dominant (Spence et al. 2008). As larger fish tend to dominate smaller ones, we were careful to select fish with similar sizes for the experiment. To reduce stress exposure for experimental fish kept in such small groups, artificial aquaria plants were added to the tanks, providing sheltering and hiding space. The exposure duration corresponds to the time necessary for the completion of two spermatogenic cycles (Leal et al. 2009) and allows for any non-genetic signals to be potentially incorporated into newly produced mature sperm (Dias and Ressler 2014; Rodgers et al. 2013).

**Fig. 1:**
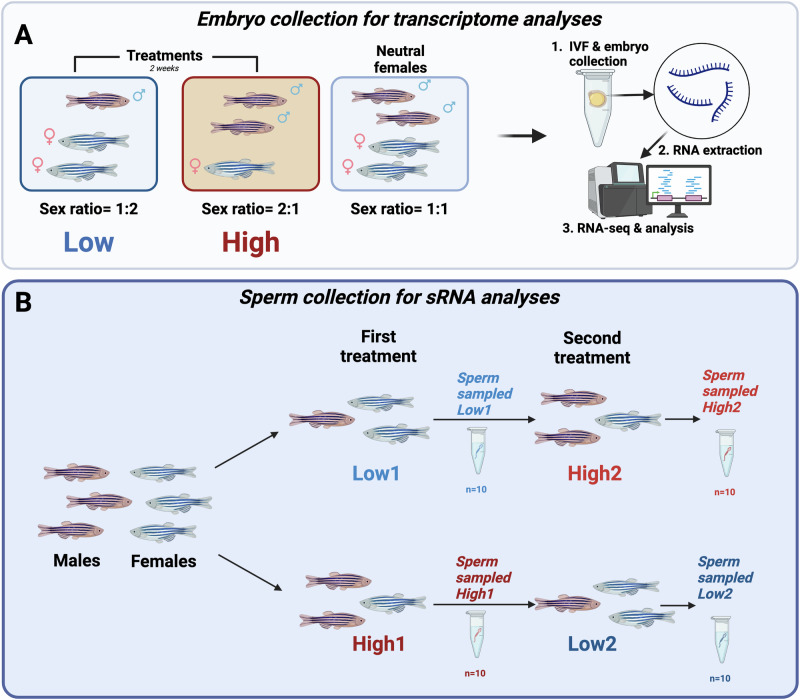
Overview of embryonic and sperm sampling in experiments. **A** Schematic of the embryonic transcriptome fish set-up, with competition environments highlighted and data acquisition in for RNA-seq experiment. **B** Schematic of the male competition environments in each treatment group for sperm sRNA sequencing experiment, namely a High competition treatment (two males and one female) and a Low competition treatment (one male and two females). All males were exposed to both treatments in randomised order with half starting High followed by Low and the other half vice versa. 1 indicates first treatment and 2 indicates second treatment. Sperm samples were collected after each treatment round for sequencing. We used *n* = 10 males in each group resulting in a total of 40 sperm samples at the two time points. Red fish are male zebrafish, and blue fish are female zebrafish.

Sperm samples were collected under anaesthesia (see Supplementary Information for details) from each male at two time points resulting in samples from High1 (High exposure first), Low2 (Low exposure second), Low1 (Low exposure first) and High2 (High exposure second) (see Fig. 1A–D for schematic of experimental set up). Each treatment lasted for 2 weeks before sperm were collected. We conducted pairwise comparisons of Low treatments only (Low1.Low2), High treatment only (High2.High1) and between Low and High (Low2.High1, Low1.High1, High2.Low1, High2.Low2).

### Sperm phenotypic data analysis

Statistical analyses of the treatment effects on sperm traits were analysed using the package *lme4* (Bates et al. 2015) for the statistical software R (see Supplementary Materials for details).

### RNA extraction, library preparation and sequencing for sperm sRNA analyses

RNA was extracted from individual sperm samples as described in Supplementary Materials. Library preparation for sRNA from sperm samples was done using the New England BioLabs kit NEBNext^®^ Multiplex Small RNA Library Prep Set for Illumina^®^ Set 1 and 2 (NEB #E7300 and NEB #E7580). The provided guidelines of NEB were followed for small sample preparation until step 15, the performing of PCR Amplification. Eight individual sperm samples were sequenced per lane on Illumina HiSeq2500 with 50 bp single-end kit resulting in an average read depth of 23 million reads per sample. Samples were combined to account for lane bias and excluding index-primer repeats. The raw sRNAseq datafiles are available on GEO here: GSE248535. Isolation of sRNA’s and quality checks can be seen in Supplementary Fig. S3 at the sequencing platform of SciLifeLab at Uppsala University.

### Experimental set-up for embryo sample collection

In this experiment, males were only exposed to one of the two different for 2 weeks, and no second treatment followed. After the 2 weeks of exposure to treatments, the sperm of experimental males were collected and used for in vitro fertilisation (IVF) procedures as described in Supplementary Material. Females used for IVFs came from separate tanks and were not exposed to any experimental treatments but kept in a 1:1 sex ratio in 3 L tanks and a density of 12 fish per tanks. These females will be referred to as neutral or control females. Embryos were collected at 24 h post-fertilisation (hpf), a key stage in early embryo development with highly synchronised expression patterns (Mathavan et al. 2005), for whole transcriptome sequencing.

### Library preparation and sequencing for embryo transcriptome analyses

RNA extraction from embryos was performed as described in Supplementary Materials. Prior to library preparation, ribosomal RNAs were depleted by using the Ribosomal RNA Depletion kit (Ambion, A1083708) following the manufacturer’s protocol. cDNA libraries were prepared from RNA samples using the NEBNext Ultra Directional RNA Library Prep Kit (Illumina compatible, NEB, #E7420L following the kit protocol, generating 125 bp paired end reads. The RNA and cDNA libraries were prepared as described above from four individual embryos from each of the 20 sub-clutches and sequenced using Illumina HiSeq 2500 Systems. The raw RNA-seq data from embryos has been deposited at European nucleotide archive accession: PRJEB66218. There was an average read depth of 15 million reads per sample. The samples were sequenced at the sequencing platform of SciLifeLab at Uppsala University.

### Bioinformatic analyses

#### Sperm sRNA analyses

Small RNA reads were converted from FASTQ to FASTA format using *seqkit* v0.15.0 (Shen et al. 2016), and then processed to trim sequencing adaptors using a custom Perl script (Fowler et al. 2019, Supplementary Material) recognising the first eight bases of the adaptor sequence (‘AGATCGGAAGAGC’).

For miRNA analyses, the trimmed reads were aligned to miRBase (v22.0) mature zebrafish miRNA sequences using *PatMaN* v1.2.2, (Prufer et al. 2008), (parameters -e 0 -g 0). A Perl script (Fowler et al. 2019, Supplementary Material) was used to parse the alignment files into a count table for each sample, which were then collated to generate an aligned read count table across all samples. *DESeq2* (Love et al. 2014) was used for normalisation of counts between samples and with treatment as the sole fixed effect to examine the effects of social stress. Adaptors for sequencing identified from FastQC reports and TrimGalore v0.6.5 were standard Illumina adaptor: AGATCGGAAGAGC.

For piRNA analyses, the trimmed reads were aligned to the piRNA reference using *PatMaN* (Prufer et al. 2008), (parameters -e 0 -g 0). A Perl script (Fowler et al. 2019, Supplementary Material) was used to parse the alignment files into a count table for each sample, which were collated to generate an aligned read count table across all samples. *DESeq2* (Love et al. 2014) was used for normalisation of counts between samples and calling differentially expressed piRNA clusters with treatment as the sole fixed effect to examine the effects of social stress. Heatmaps of log^2^ fold change for differential expression of miRNAs and piRNAs were generated using the heatmap package version 1.0.12 in R Studio (Kolde 2012).

### Differential gene expression analyses in embryos

We sequenced a total of 80 embryos with four embryos per sub-clutch from a total of 20 sub-clutches from ten males (five per treatment) and ten females. The analysis of the RNA-sequencing data was conducted following (Conesa et al. 2016). Quality control was assessed using FastQC v0.11.6 (Andrews 2010). Reads were mapped against the zebrafish reference genome version (Genome Reference Consortium 2017) and counted at the gene level using STAR v2.5.3a (Dobin et al. 2013). Differential gene expression was analysed with the R package *DESeq2* v1.34.0 (Love et al. 2014) with treatment and female ID as fixed effect. We used R packages *EnhancedVolcano* v1.12.0 for plotting volcano plots (Blighe et al. 2021). ShinyGO v0.77 was used for GO terms analysis with a custom specified background list of all genes expressed with a *p* value in the *DESeq2* output results.

### Sperm piRNA analysis

Zebrafish GRCz11 piRNA clusters were downloaded from piRNA Cluster Database (Rosenkranz 2016) in bed format and GRCz11 reference assembly genome was downloaded from NCBI Genomes (https://www.ncbi.nlm.nih.gov/datasets/genomes/?txid=7955). A total of 532 piRNA cluster loci were extracted from the reference assembly genome using the genomic coordinates of the piRNA clusters in the .bed file using the *bedtools* (v2.29.2) function *getfasta*, producing the reference sequence for piRNA clusters. PiRNA loci not found in the NCBI reference assembly (e.g. loci in scaffolds/contigs) were not included.

### De novo sperm piRNA cluster generation

Small RNAs were adaptor trimmed using *trimgalore!* (v 0.6.6, --stringency 8), (Krueger 2019), with cutadapt v1.18 (Martin 2011). Trimmed sRNAs were then converted from fastq to fasta format using seqkit fq2fa (v0.15.0) (2) all sequencing files where concatenated into a single processed sRNAs fasta file for piRNA cluster prediction. To identify additional piRNA clusters not found in the piRNA cluster database, we ran the *proTRAC* pipeline v 2.4.4 on the fasta file of piRNAs generated as described above (Rosenkranz and Zischler 2012). All sequencing files where concatenated into a single processed sRNAs fasta file for piRNA cluster prediction. In brief, the script TBr2_collapse and TBr2_duster was used to remove redundant and low-complexity sequences from the processed sRNAs. sRNAs were then mapped using the perl script *sRNAmapper* v1.0.5 (Roovers et al. 2015), to the *Danio rerio* genome GRCz11. The resulting map file was then processed with the Perl script proTRAC_2.4.4 to predict piRNA clusters. In total, we discovered 902 new piRNA clusters concatened with the existing 532 piRNAs from the reference genome, to generate a new piRNA reference with 1434 piRNA clusters in total. Locus identifiers within the expression tables and results table starting with NC_are previously identified clusters, all others are de novo identified with proTRAC. PiRNA loci located on unplaced scaffolds/contigs were not included.

The resulting fasta file containing piRNA clusters was concatenated with the existing piRNA database and used as the set of piRNA reference sequences called from this point onwards the ‘piRNA reference’ (1434 piRNA clusters in total). Locus identifiers within the expression tables and results table starting with NC_ are clusters from the existing database, all others are de novo identified with *proTRAC*.

### Differential expression of embryo TEs

To identify differentially expressed TE families between the embryos of High and Low competition males, we used TEtranscripts v2.2.3 on the transcriptome data (Jin et al. 2015). The TEtranscripts pipeline was run using -g GRCz11 genome settings. Strandedness of our RNA-seq data was checked with MultiQC report (Ewels et al. 2016). TEtranscripts was executed with the following settings: *sorted_bam_files.bam --GTF Danio_rerio.GRCz11.107.gtf --TE GRCz11_Ensembl_rmsk_TE.gtf --mode multi --project projectname --minread 1 -i 100 --padj 0.05 --outdir results*.

### Sperm miRNA target gene analysis

For sensitivity, statistical confidence of miRNA-target gene associations in the 3’ untranslated region (UTR) of mRNAs by miRNAs, miRanda v3.3a pipeline was used with default settings (John et al. 2004). MiRanda was run with fasta file of mature zebrafish miRNAs and 3’ UTRs to scan and generate scored putative matches in the output. Full scripts and files can be accessed here: https://github.com/alicegodden/paternalsocstress/tree/main/miRanda. Zebrafish UTRs were obtained from TargetScan (Agarwal et al. 2015) and the mature miRNA fasta file was obtained from miRbase (Griffiths-Jones et al. 2006). The *ShinyGO* web-tool v0.77 was used for GO terms analyses on target genes (Ge et al. 2020). The background list of genes for sperm was taken from published scRNAseq datasets (Qian et al. 2022) and was limited to genes known to be expressed in sperm. To find and identify piRNAs in the differentially expressed clusters, the genomic regions were searched with piRBase (Wang et al. 2019).

To test for a link between sperm piRNAs and embryo TE, we used *FishPi*, a Python-based (*Python* v3.11) piRNA-TE complementarity analysis tool (Godden et al. 2025). *FishPi* works by matching piRNA seed regions specified by user (1–10 bp for 5’ end of mature piRNA for teleost) as input, and TE matches are identfied in a reference fasta file containing a list of all known TE sequences.

To link gene expression and TE expression in the embryo dataset, we developed Fish Transposable Element Analyser (*FishTEA*). We compared overlapping significantly differentially expressed genes and significantly differentially expressed TE loci using the DanRer11/GRCz11 reference genome. All scripts were written in Python v3.11. *FishTEA* works in five key steps that are ran individually: step 1: addition of TE loci co-ordinates, 2. Matching overlapping genes and TEs, 3. Generation of a chromosomal plot, 4-5—plotting bar charts at TE family / class level count data. 4. Hypergeometric testing for enriched overlapping regions of TEs and genes in the genic region of the genome. The aim was to visualise any overlaps between significantly differentially expressed genes and significantly differentially expressed TEs and to identify particularly active regions in the genome. *FishTEA* can be easily adapted to other organisms and can be found on Github: https://github.com/alicegodden/FishTEA/.

All scripts used in this project can be found here unless otherwise specified: https://github.com/alicegodden/paternalsocstress/.

## Results

### Phenotypic sperm traits

To test for the effect of competition environment on sperm traits, we compared velocity parameters of sperm between High and Low males. We found a significant difference in VCL (curvilinear velocity) in sperm between males from the High (HighDom and HighSub Supplementary Fig. 1), and the Low competition (LowDom and LowSub Supplementary Fig. 1) treatments where Low competition males produced faster sperm than High competition males. Statistical parameters came from a linear mixed model (REML) with an Analysis of Deviance (Type III Wald chi-square tests, Treatment:Time *p* = 0.0315359, social status: Treatment:Time:Status *p* = 0.0009481 (see Supplementary Fig. S1 and Supplementary Table S1 for VCL and full statistical analyses).

### Differential expression of sperm miRNAs

**Fig. 2:**
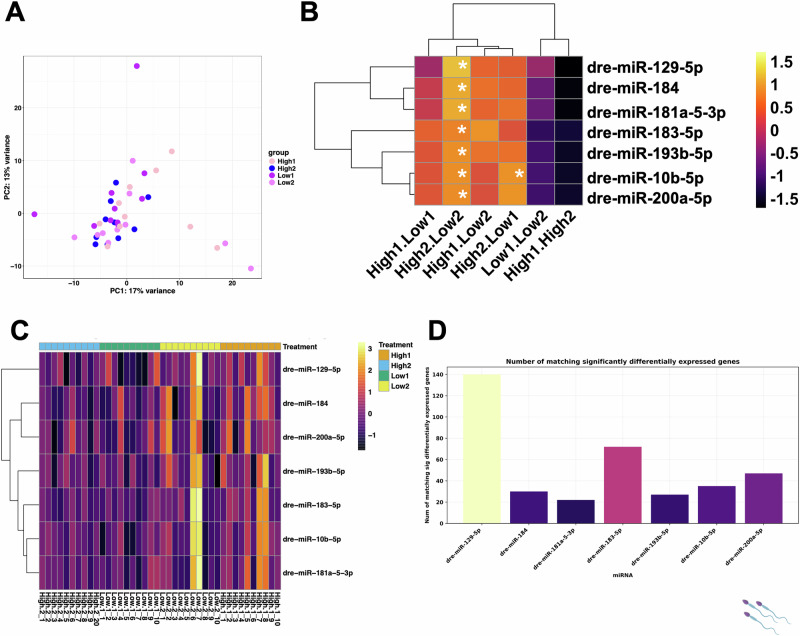
Differential expression of miRNAs in sperm of males exposed to High and Low male-male competition environments. **A** Principal component analysis showing variation across all samples used in the miRNA differential expression analysis. **B** Heatmap with dendrogram clustering showing all statistically significantly differentially expressed miRNAs log^2^ fold change (*p* = <0.05 denoted by *). Colour key reflects miRNA expression level, and shows general trend of miRNA expression. Not all miRNAs were significantly differentially expressed across all groups. **C** Heatmap of normalised counts (log^2^ CPM), from DESeq2 output of sRNA-seq data on miRNAs across all samples, grouped by treatment. **D** Number of matching significantly differentially expressed genes that targeted a significantly differentially expressed miRNA. 515 genes targeted the miRNAs.

We compared the miRNA profiles of males exposed to both High and Low treatments. In a first step, a principal component analysis across all samples including treatment and the unique Male ID assigned to every male at the start of the experiment showed a substantial effect of male ID on sperm miRNA profiles (Fig. 2A). Nevertheless, we identified a total of seven significantly differentially expressed miRNAs across the pairwise comparisons between the four treatment groups (Fig. 2B), one of which was found in the High2.Low1 comparison (*dre-miR-10b-5p*). This miRNA and the other 6: *dre-miR-129-5p*, *dre-miR-184*, *dre-miR-181a-5-3p*, *dre-miR-183-5p*, *dre-miR-193b-5p*, *dre-miR-10b-5p*, *dre-miR-200a-5p* were all significantly differentially expressed in the High2.Low2 pairwise comparison. No significantly differentially expressed miRNAs were found in any other pairwise comparison.

When looking at log_2_ transformed counts per million (CPM) *Z*-scores of significantly differentially expressed miRNAs across all samples (Fig. 2C), miRNA expression showed minimal variance across samples, with Low2_6 and Low2_7 showing higher expression for all miRNAs. Low1 samples appeared to show the lowest expression of miRNAs, whereas High1 showing the highest expression of miRNAs. Following high social stress environments the miRNA dre-miR-129-5p targets 140 significantly differentially expressed genes in the embryonic transcriptome dataset (Fig. 2D).

### Differential expression of sperm piRNA clusters

**Fig. 3:**
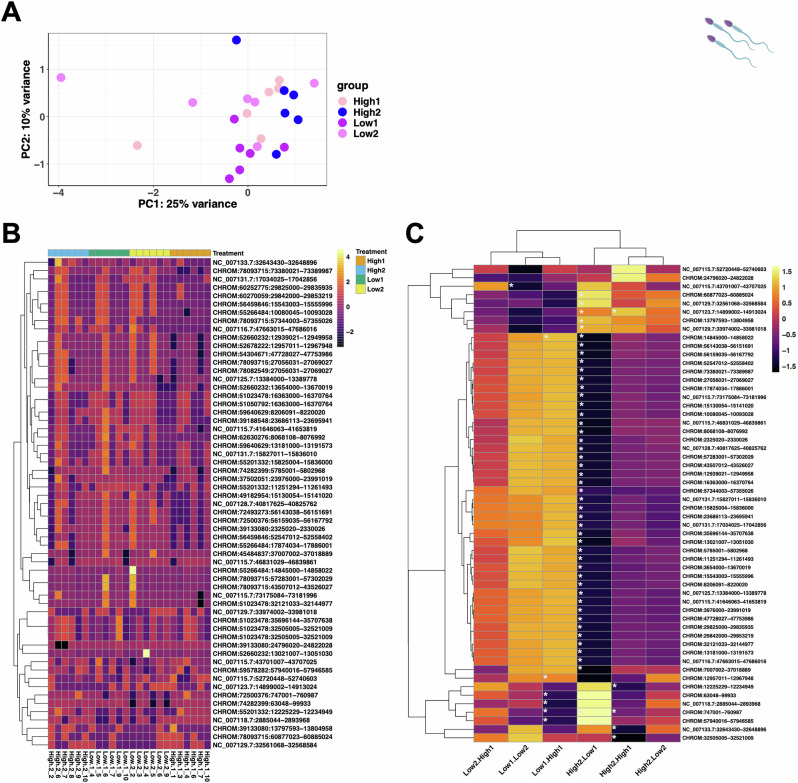
Differential expression of piRNAs in sperm of males exposed to High and Low male-male competition environments. **A** Principal component analysis showing variation across all samples used in the piRNA differential expression analysis. **B** Heatmap of normalised counts (log^2^ CPM) from DESeq2 output of sRNA-seq data on piRNAs across all samples, grouped by treatment. **C** Heatmap of all significantly DE piRNAs clusters log^2^ fold change (*p* = <0.05 are denoted by *) for pair-wise comparisons. Colour key shows piRNA expression level, and shows general trend of piRNA expression. PiRNA names are abbreviated (see Supplementary File S1 for full piRNA cluster loci names).

We compared the piRNA profiles of males exposed to both High and Low treatments. A principal component analysis of piRNAs showed clear clustering by Male ID and some clustering by treatment groups on PC1, with Low competition treatments clustering with some degree of separation from the High competition treatments (Fig. 3A). We found a total of 1434 piRNA clusters in sperm, of which 532 were already known (Fig. 3B, C), and 902 were unknown de novo clusters from the *proTRAC* output. We observed a general increase in expression of piRNA clusters in sperm samples of all Low males (Low1 and Low2) compared to all High males (High1 and High2; Fig. 3C). However, we found significant differences when comparing the different treatments according to experimental order (High1 and High2 and Low1 and Low2). The most significantly differentially expressed piRNA clusters were found when comparing sperm samples from High2 and Low1 males, piRNAs in this comparison were mostly downregulated in expression. The most upregulated piRNA cluster in this comparison was *NC_007123.7:14899002-14913024* and the most downregulated clusters included many unannotated piRNA clusters from *CHROM:14845000-14858022* to *CHROM:7007002-37018889*. A significant trend of downregulation of piRNA clusters was also observed in the comparison of groups Low1-High1, and High2-High1. The general pattern across these comparisons was the significant downregulation of piRNA clusters in sperm of males exposed to a High treatment (Fig. 3B).

Significantly differentially expressed piRNA read counts that were log_2_ transformed CPM with *Z*-scores showed higher piRNA expression in the High2 group, and lower overall expression in the High1 group (Fig. 3B). When looking for differential expression in piRNA clusters between treatments (Fig. 3C), we identified 45 differentially expressed piRNA clusters between samples from the same males in the comparison Low1.High2 (Fig. 3C, denoted by asterisks). Of note, there were no significantly differentially expressed piRNAs for the Low2.High1 and High2.Low2 comparison of samples. However, we found six differentially expressed piRNAs (Fig. 3C) in the Low1.High1 comparison, five differentially expressed piRNA clusters in the High1.High2, and one differentially expressed piRNA in the Low1.Low2 comparison (Fig. 3C).

**Fig. 4:**
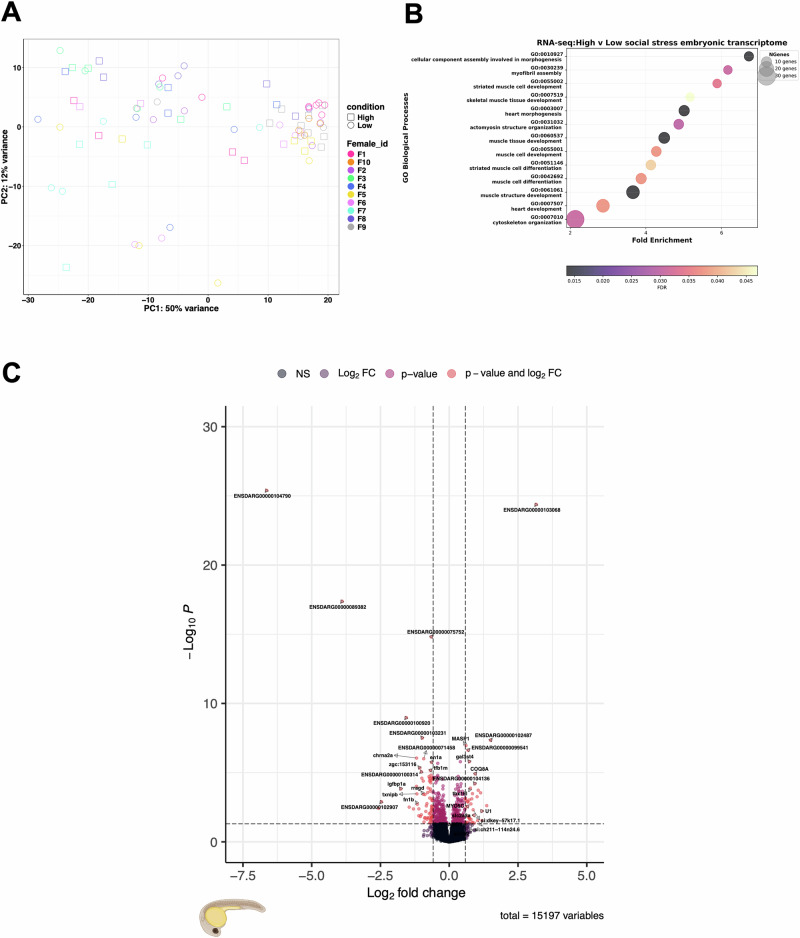
Differential expression of mRNAs in 24 hpf half-sibling embryos sired by males exposed to High and Low competition treatments. **A** Principal component analysis with treatment (Low versus High), and female ID as factors from DESeq2. **B** GO term analysis looking at biological processes for zebrafish using ShinyGO on all significantly differentially expressed genes (FDR cut-off was set to *p*_adj_ < 0.05) revealed a significant prediction to affect muscle development and differentiation. **C** Enhanced volcano plot of differentially expressed genes from DESeq2.

According to piRBase (Wang et al. 2019), piRNA cluster *CHROM: 57940016-57946585* is located on chromosome 1 and contains 125 known piRNA sequences within that cluster. This cluster was significantly downregulated in comparison Low1.High1. The largest identified piRNA cluster is located on chromosome 4, *NC_007115.7:52720448-52740603* at 20.16 kb in size. It contains the sequences of 169 known piRNAs, and was significantly enriched in sperm from High competition males, showing the highest fold change in expression. The *NC_007133.7:32643430-32648896* cluster is located on chromosome 22, is 5.4 kb in size and contains six known piRNA sequences, and three ncRNA genes: *BX640547.5*, *BX640547.4*, *BX640547.6*. This piRNA cluster was significantly downregulated in sperm from High competition males (High1 and High2) (Fig. 4B). From the largest piRNA cluster, *NC_007115.7:52720448-52740603*, individual piRNAs were further analysed with *FishPi* to identify matching TE transcripts. As there were 169 piRNAs in this cluster, the first piRNAs of the three biggest subclusters were selected for analysis (Supplementary Fig. 5). The most significant and complementary class of TEs to *piR-dre-43599*, *piR-dre* -*7058* and *piR-dre*-*58396* were class I DNA transposons, with fewer complementary class II RNA retrotransposons (Supplementary Fig. 5). *piR-dre-43599* was complementary to 2080 TEs (1846 Class I and 234 Class II—Supplementary Fig. 5A), *piR-dre -7058* was complementary to 1160 TEs (874 Class I and 286 Class II—Supplementary Fig. 5B) and *piR-dre-58396* was complementary to 2483 TEs (1943 Class I, 540 Class II—Supplementary Fig. 5C).

### Embryonic transcriptome analysis

#### Differential gene expression in 24hpf embryos

To assess an effect of the competition environment in fathers on offspring gene expression, we compared the transcriptome of embryos sired by either High or Low fathers in IVFs performed after 2 weeks of treatment. We identified a total of 612 differentially expressed genes with a *p*_adj_ < 0.05 between 24 hpf embryos sired by High and Low competition males (see Supplementary Files 3 and 4 for raw RNA-seq counts data and associated metadata). A principal component analysis showed negligible clustering of expression profiles by treatment groups or maternal effects (Fig. 4A). Of the differentially expressed genes, 358 were downregulated and 254 were upregulated in High competition embryos. *ENSDARG00000104790* was the most downregulated differentially expressed gene in High competition embryos (Fig. 4C) and *Metazoa_SRP* (*ENSDARG00000103068*) was the most upregulated differentially expressed gene (Fig. 4C). GO terms analyses on biological processes showed strong associations of these two gene groups with muscle development and differentiation (Fig. 4B). *ENSDARG00000104790* (*CABZ01045617*.1) is expressed in the vascular system in zebrafish embryos (Gurung et al. 2022). *Metazoa_SRP* (ENSDARG00000103068), which is approximately 300 bp long (cDNA) and is present in multiple copies (81 hits on Ensembl) in the zebrafish genome, with most copies located on chromosome 11. These are necessary components for co-translational protein targeting by the signal recognition particle (Bradshaw and Walter 2007; Keenan et al. 2001).

### Social stress impact on TE expression in the embryonic transcriptome

**Fig. 5:**
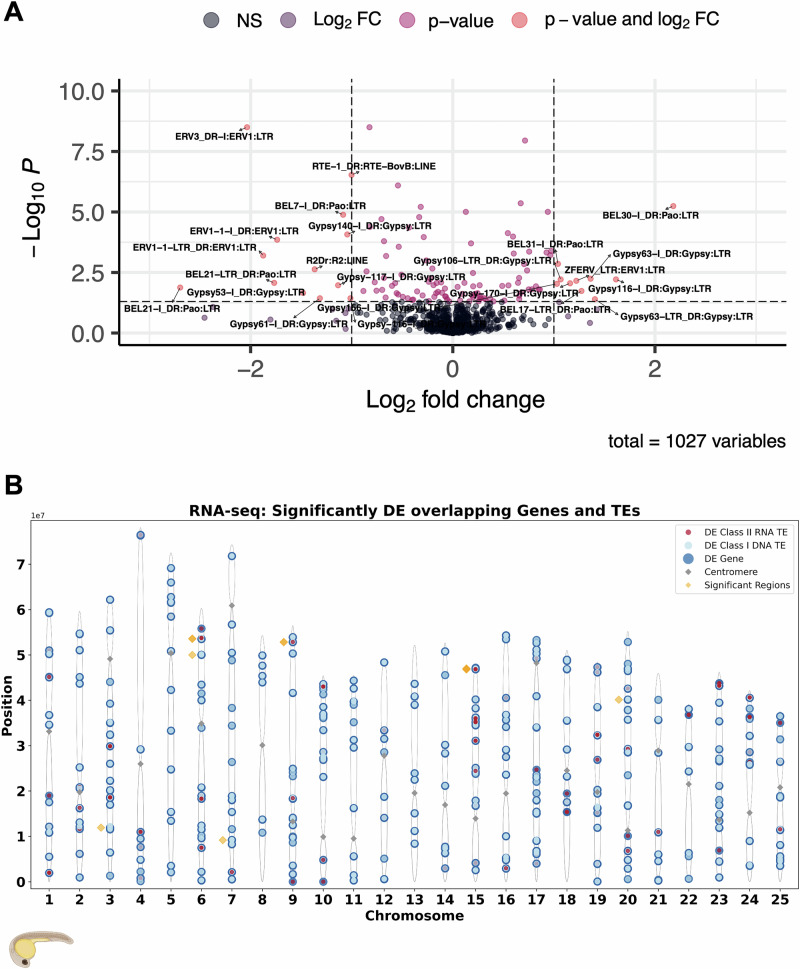
Differential expression of TE transcripts by class and family in embryos fathered by males from High versus Low competition environments. **A** Volcano plot of differentially expressed TEs by family from *DESeq2* analyses looking at impact of high social stress and factoring in female_id. **B** Chromosomal distribution of the significantly differentially expressed genes and TEs that overlap along each chromosome with enrichment testing. Differentially expressed genes (dark blue), Differentially expressed TEs Class I (red), Differentially expressed TEs Class II (light blue). Regions where the overlap of Differentially expressed genes and differentially expressed TEs in genic regions is statistically significant—are highlighted in orange diamonds. In total there are 12 significant regions of enrichment out of a possible 1898.

To test for an effect of the competition environment experienced by fathers on TE activity, we compared TE expression in embryos sired by either High or Low fathers in IVFs performed after 2 weeks of treatment. The embryo transcriptome data were analysed with the *TEtranscripts* pipeline (Jin et al. 2015) to profile differential expression of TEs at the family level (Fig. 5A). A principal component analysis of the TE data showed modest clustering of the samples according to the two treatments and female ID (Supplementary Fig. S4A). The most significantly differentially expressed TEs at family level were ERV, BEL and Gypsy TEs, which belong to the LTR class (Fig. 5A). The most abundant TEs were Class I DNA TEs (Supplementary Fig. S4B, C). We used *FishTEA* (Godden and Immler 2024) to test if TEs overlapped with genes that were differentially expressed and to reveal regions of the genome showing enrichment or downregulation of gene expression or TE activity. We found 343 (out of 612) significantly differentially expressed genes to overlap with 59 (out of 116) significantly differentially expressed TEs. We found that the majority of these TEs were Class I DNA TEs, as denoted by the orange dots, with a few Class II RNA retrotransposons, as denoted by the blue dots (Fig. 5B). Regions of highest enrichment for shared clustering of TEs and differentially expressed genes included the short arm of chromosome 4 and chromosome 23. Interestingly, the long arm of chromosome 4 showed little activity of significantly differentially expressed TE families and genes. Regions with significant enrichment of significantly differentially expressed genes and TEs in genic regions are highlighted in orange diamonds, we found 12 of 1898 regions where our genes and TEs overlapped were significant (*p* = < 0.05) (Fig. 5B).

### Linking sperm sRNAs with DE genes in embryos

To link the significantly differentially expressed genes identified in the embryos, and the significantly differentially expressed miRNAs in the sperm, we used *miRanda*, which identifies the genes targeted by specific miRNAs by searching for complementary hits in the 3’ UTR. The number of genes that are targeted by specific miRNAs are referred to as hits: *dre-miR-129-5p* had 140 hits, *dre-miR-184* had 30 hits, *dre-miR-181a-5-3p* had 22 hits, *dre-miR-183-5p* had 72 hits, *dre-miR-193b-5p* had 27 hits, *dre-miR-10b-5p* had 35 hits and *dre-miR-200a-5p* had 47 hits (Fig. 3D). The 3’UTR’s predicted to be the top hit of the significantly differentially expressed miRNAs showed 515 (out of 612) of the significantly differentially expressed genes as predicted targets (Supplementary Fig. S2H). We performed GO terms analysis to examine potential affected biological processes and found enrichment for terms associated with muscle development terms (Supplementary Fig. S6).

We found 224 out of 612 significantly differentially expressed genes from our embryonic transcriptome data were targeted by the 7 significantly differentially expressed miRNAs from our sperm data, as predicted by miRanda (Supplementary Fig. S2A–G. In sperm from High2 males, the miRNA *dre-miR-129* was enriched (Fig. 2B) and was predicted to target significantly downregulated genes *ENSDARG00000075752* (*MYO18A*) and *ENSDARG00000103231* (novel gene) in the embryos (Supplementary Fig. 2G). A Fisher’s exact test to examine for enrichment of miRNA target genes in the differentially expressed genes from embryos, showed that the differentially genes targeted by sperm miRNAs were highly enriched over random (odds ratio = 129.26; *p* = 2 × 10^−16^).

## Discussion

Variation in male-male competition not only affected sperm velocity parameters but also sperm sRNA, TE and gene expression profiles. Environmental changes and stress may affect the defense mechanisms in the male germ line where small RNAs play a key role in the defense of the genome against the activity of TEs and other possible DNA-damaging elements (Godden and Immler 2023). These same signals may induce a potentially mild stress response in the resulting offspring, which affects early growth and development. Use of *IVF* in the externally fertilising zebrafish made our results powerful and allowed us to focus more on paternal effects while controlling for maternal effects (Mashoodh et al. 2018). Here, we discuss how a response to competitive male social environments may explain the changes in mi- and piRNA profiles in sperm and how these changes may be linked to perturbed gene and TE expression differences in the embryo RNA-seq data.

As previously described in many taxa (Bonduriansky and Crean 2018; Zajitschek et al. 2014, 2017), the level of male-male competition did have an effect on sperm velocity where low competition males produced faster sperm than High competition males (Supplementary Fig. S1 and Supplementary Table S1 for VCL). This confirms that our treatments had the expected effects in response to male social environments, and that high competition males experienced more stress reducing overall sperm performance.

### Paternal stress effects on gene expression in zebrafish embryos

We found a significant general enrichment of GO terms for muscle development in High competition embryos (4B). Specifically, T-box transcription factor 16-like gene *tbx16l* was enriched in embryos sired by males under High competition environments, a which is involved in mesoderm and somite development (Windner et al. 2015). An increase in expression of *tbx16l* could lead to more rapid muscle development and earlier onset of larval activity. Other significantly differentially expressed genes involved in embryo development include *si:dkey-121a11.3, myo18a* and *masp1*. Differentially expressed genes involved in the development of the central nervous system include *masp1*, *chrna2a* and *ern1*, and *en1a*. Finally, *gal3st4, ano7* and *coq8ab* are involved in general metabolic pathways and lipid metabolism. The GO terms for biological processes for miRNA revealed enrichment of processes in metabolism, muscle and nervous development (Supplementary Fig. 5). All these processes indicate that embryos may differ in their developmental and metabolic rates, which links back to the earlier finding of increased hatching rates in embryos sired by High completion males (Zajitschek et al. 2014), and highlights potential detrimental effects due to poor male condition in a high competition environment (Bonduriansky and Crean 2018). Overall, we show that the response in males exposed to a High competition environment is translated into the offspring and triggers similar physiological responses. Such a response has been observed in offspring of starved zebrafish males where the gene expression in the embryos reflected gene expression changes observed in starved adult zebrafish (Jimenez-Gonzalez et al. 2024).

### Effects of paternal stress on TE activity in embryos

TE activity was altered at the family level, with strong enrichment and downregulation of TE families in High competition embryos (Fig. 5A). A possible explanation for the effects we observed is that males facing a rival male may have a physiological response to the higher stress levels induced by the competitive environment (Bonduriansky and Crean 2018; Filby et al. 2010). This response affects the sRNAs in the sperm and their transfer into the zygote may explain the differential gene expression observed in the embryos as well as the differences in TE activity. Additionally, we found that 343 significantly differentially expressed genes overlapped with loci of 59 significantly differentially expressed TEs (Fig. 5B). These TEs may be hitchhiking the gene expression mechanisms to upregulate themselves, and impact gene expression, with two-thirds of zebrafish TEs known to be associated with host gene expression (Chang et al. 2022). Such detrimental TEs would be expected to be selected against and removed quickly over evolutionary times and time-point sampled transgenerational studies will give more insight into directional selection.

### Sperm miRNAs

sRNAs are known to be involved in inter-and trans-generational inheritance of parental conditions (Duempelmann et al. 2020; Rechavi and Lev 2017). In zebrafish, at least 415 miRNAs are known, which are split into 44 families, with *miR-430* being the largest family with many isoforms; *miR-430* is located on chromosome 4, which has the least protein-coding genes of all zebrafish chromosomes (Howe et al. 2013; Thatcher et al. 2008). We found *miR-10b-5p* to be the most significantly differentially expressed miRNA in sperm of High versus Low competition males (Fig. 2B). This miRNA is highly conserved across the animal kingdom (Griffiths-Jones 2004; Griffiths-Jones et al. 2006). In house mice, *miR-10b* is involved in spermatogonial stem cell production and loss of *miR-10b* may lead to increased spermatogonial stem cell death (Li et al. 2017). *MiR-10b* is targeting *Kruppel-like factor 4* (*Klf4*), a transcription factor that is involved in cell cycle regulation, contact inhibition and apoptosis, and is highly expressed in germ cells and Sertoli cells (Li et al. 2017). A possible explanation is that the high competition treatment led to changes in sperm production by deregulation of sperm stem cell renewal as shown in dominant zebrafish males that have higher numbers of spermatids in their testes in comparison to subordinate males (Filby et al. 2010). The trend of enrichment of miRNAs in response to environmental stress may represent an adaptive mechanism to fine-tune gene expression and preserve the germline genome (Godden and Immler 2023).

Physiological stress affected sRNA profiles in sperm following heat-stress in *Drosophila* where genes coding for heat shock proteins, including *Hsp68* and *Hsp70*, became highly enriched, and some sRNAs (miRNAs and piRNAs), and Gypsy family TEs were differentially expressed (Bodelon et al. 2023). Similarly, *Drosophila* sperm miRNA profiles changed in response to variation in population sex ratio, where *miR-184* was found to be differentially expressed (Hotzy et al. 2021). In fruitflies, *miR-184* is important in the production of the female germline (Iovino et al. 2009) and given that mature *miR-184* is conserved between zebrafish and fruitflies, it may have shared functions in germ cell development (Zhang et al. 2023). Other differentially expressed miRNAs in our study included *dre-miR-129-5p, dre-miR-181A-5-3p and dre-miR-193B-5p*, (all enriched in High2.Low2). *Dre-miR-129* is conserved across vertebrates and is involved in zebrafish ciliogenesis, with inhibition of *dre-miR-129-3p* showing defective embryo development and suppression of ciliation in the Kupffer’s vesicle (Cao et al. 2012). *Dre-miR-181A-5-3p* is involved in zebrafish vascular development where experimental over-expression and knock-down impairs blood vessel development (Ma et al. 2019). Additionally, *miR-181A-5p* is known to regulate inflammatory immune responses and is involved in tumour development (Ye et al. 2018). Finally, *dre-miR-193B-5p* was found to be significantly downregulated in zebrafish larvae exposed to 1% ethanol (Soares et al. 2012). Stress induced by the high competition environment may therefore affect immune response and cardiac and developmental pathways. *Dre-miR-200* was enriched in High versus Low comparison groups, most significantly in High2.Low2. The *dre-miR-200* cluster is involved in the regulation of sperm motility, with increased expression leading to decreased sperm motility by targeting sperm-motility genes including *amh* (Xiong et al. 2018).

To link sperm miRNA with the embryo transcriptomes, the significantly differentially expressed miRNAs were put through miRanda to analyse predicted gene targets (Supplementary Fig. S2). Of the 612 significantly differentially expressed genes from our embryonic RNA-seq data, 515 were targeted by our miRNAs, with each miRNA targeting some significantly differentially expressed genes, enrichment testing with Fisher’s exact test showed *p* = 2.2 × 10^−16^ with an odds ratio of 129.256. This shows a strong enrichment of the miRNA target genes (Fig. 2D). Each of these miRNAs were enriched following their competition environment treatment, in the High2 v Low2, High2 v Low1 comparisons (Fig. 2B).

### Linking sperm piRNAs and embryo TEs

PiRNA enrichment helps to maintain the integrity of the germline through complementary binding to transposon transcripts, whereas loss of piRNAs leaves the germline genome vulnerable to invasion and disruption by transposons (Aravin et al. 2007). Chromosome 4 is the putative sex chromosome in zebrafish and hosts genes that can influence sex determination, particularly around position 61,176,889 (Anderson et al. 2012; Howe et al. 2013), which may explain the over-representation of repetitive elements in that region. We found the largest differentially expressed piRNA cluster *NC_007115.7:52720448-52740603* on chromosome 4 to be enriched in High competition sperm (Fig. 3B, C), which supports the idea that piRNA expression is linked to a response in the germ line to environmental stress to protect the germline genome (Godden and Immler 2023; Klattenhoff and Theurkauf 2008). No significantly differentially expressed genes and overlapping TEs on a significant portion of the long arm of chromosome 4; a region that is known to be particularly rich in piRNAs and in retroelements (Chang et al. 2022; Howe et al. 2013).

Approximately 56–59% of the zebrafish genome consists of TEs (Shao et al. 2019), and the majority are DNA transposons covering 46% and RNA retrotransposons 13% respectively (Chang et al. 2022). We found LTR elements to be most significantly differentially expressed between High and Low embryos (Fig. 5A). LTRs are most enriched during early embryonic developmental stages (Chang et al. 2022), which may explain a bias towards the significantly differentially expressed LTR elements in our study. Most zebrafish piRNAs map to TEs (Houwing et al. 2007), where a majority map to LTR elements, and fewer piRNAs map to DNA transposon targets, suggesting that piRNA changes with the environment to maintain the integrity of the germline genome (Chang et al. 2022). Our results suggested substantial complementarity of some of piRNAs in the differentially expressed cluster *NC_007115.7:52720448-52740603* to DNA TEs (Supplementary Fig. 6). DNA TEs are linked to older TE age and events (Chang et al. 2022) and retained activity correlated with longevity in *C*. *elegans* (Sturm et al. 2023). Therefore it is expected that piRNAs complementary to the LTR elements are responding to increased TE activity from the environmental stressor.

In the zebrafish genome, LTRs and LINEs are mainly located in pericentromeric regions whereas DNA transposons are located near or within genes; with over 60% of TE transcripts thought to be driven by local gene promoters (Chang et al. 2022). In the adult zebrafish genome, 37% of TEs are located in active regulatory states (Lee et al. 2022). In accordance, we found significantly differentially expressed TEs from Class II retroelements in the pericentromeric region, and Class I DNA TEs overlapped with gene loci more abundantly and frequently than Class II elements (Fig. 5B and Supplementary Fig. 6). This finding suggests that DNA TEs may be expressed more readily, and co-expressed with genes expression responding to the High competition environment.

Many piRNA functions remain unknown, and most but not all piRNAs currently match transposon sequences. In addition, multiple piRNAs may target the same or different TE mRNAs (Zhang et al. 2018). Therefore, continued development of bioinformatic tools such as FishPi (Godden et al. 2025), FishTEA (in this paper), and CRISPR of TEs (Guo et al. 2024) as well as functional tests by knocking out mi- and piRNAs are needed to further analyse the function and interactions of sRNAs and TEs to further understand the mechanisms of their response to environmental triggers.

### Social dominance and environment change

When running pairwise comparisons of sperm sRNAs between specific treatment groups (Low1, Low2, High1, High2), we identified a number of differentially expressed mi- and piRNAs that varied across comparisons When comparing Low1 and Low2 only, we observed general downregulation of miRNAs and enrichment of piRNAs, whereas when comparing High2 and High1, we observed downregulation of miRNAs and piRNAs. In sperm from males in a Low treatment followed by High exposure, we found modest enrichment of miRNAs and some enrichment and downregulation of piRNAs. In sperm of males exposed to High competition, we found modest enrichment of miRNAs and downregulation of piRNAs. Overall, exposure to the High competition treatment seemed to have the most profound effect and led to significant changes in sRNA expression, and males exposed to High competition first and then Low competition differed from males, that had only been exposed to a Low competition environment. Nevertheless, the environment a male was exposed to immediately before sperm collection had the strongest effect on the direction of mi- and piRNA expression. It appears therefore that the history and specific sequence of environments affects several spermatogenic cycles. Spermiogenesis takes approximately 1 week in zebrafish (Leal et al. 2009), so having had two cycles of 2 weeks the second sperm sample is still showing some signs of impact from the first environmental exposure.

Thermal stress environments have previously been shown to reduce male fertilisation success in zebrafish, with increasing thermal stress negatively affecting sperm quality and subsequently fertility (Irish et al. 2024). Similarly, alarm cues affect sperm sRNAs in zebrafish supporting the idea that the paternal environment may influence offspring phenotype, via paternal inheritance of sRNAs (Ord et al. 2020). Our study showed that 224 of 612 significantly differentially expressed genes were also predicted targets of the significantly differentially expressed miRNAs. Together, this highlights the importance of the role of miRNAs for paternal inheritance.

## Conclusion

We provide clear evidence that variation in the social environment of zebrafish males can affect sRNA expression in their sperm and gene TE expression in the resulting offspring. We provide direct links between sperm sRNAs and differentially expressed genes in the embryos by introducing new bioinformatic tools and link the seven significantly differentially expressed sperm miRNAs to a global regulatory function over the 612 significantly differentially expressed genes in the embryos. Overall, we provide links between genes and TEs (FishTEA), genes and miRNAs, piRNAs and TEs (FishPi). The regulatory functions of many of the differentially expressed genes are in line with the general phenotypic effects described in previous studies (Zajitschek et al. 2014, 2017). We are still at the very beginning of understanding the potential effects of paternal condition on offspring fitness, but our study further supports the idea that variation in paternal condition, even over short periods of time, is likely to have a significant effect. We currently can only speculate about the mechanisms responsible for the transfer of signals about paternal condition from father to offspring (Immler 2018; Krawetz 2005) and understanding these mechanisms certainly deserves more attention in the future. Whether the epigenetic changes caused by a environmental variation are a true inheritance factor or rather a marker of underlying mechanisms defending the germ line genome (or both) remains to be further investigated.

## Supplementary information

Supplementary files for Godden et al. 2025

Supplementary material

Supp File 1

Supp File 2

Supp File 3

Supp File 4

Supp File 5

Supp File 6

Supp File 7

Supp File 8

Supp File 9

Supp File 10

## Data Availability

The raw sRNAseq datafiles are available on GEO: GSE248535, http://www.ncbi.nlm.nih.gov/geo/query/acc.cgi?acc=GSE248535. The raw RNA-seq datafiles are available on the European Nucleotide Archive (ENA, http://www.ebi.ac.uk/ena/browser/view/PRJEB66218). All metadata, raw counts data, and scripts used to generate and visualise the results used in this project are available as supplementary files in the Supplementary Materials.
