# Supplementary material for: Environmentally induced variation in sperm sRNAs is linked to gene expression and transposable elements in zebrafish offspring
Source: Heredity (Edinb). 2025 Mar 22;134(3-4):234–46. doi: 10.1038/s41437-025-00752-2 (PMC11977266; doi:10.1038/s41437-025-00752-2)
Supplement: Supplementary file 1 — Supplementary files for Godden et al. 2025 [file 41437_2025_752_MOESM1_ESM.docx]

**Supplementary Files**

**Supplementary materials, methods and figures and figure legends**

Supplementary Material_heredity.docx

**Supplementary File S1- List of piRNA cluster full names in Fig. 4B**

Supp_File_1_piRNAs_heatmap_fullnames.xlsx

**Supplementary File S2 – Phenotypic data**

Supp_File_2_sperm_motility_data.csv

**Supplementary File S3 & S4- raw counts and metadata file RNA seq**

Supp_File_3_RNA_seq_rawcounts.csv

Supp_File_4_rnaseq_metadata.csv

**Supplementary File S5 & S6- raw counts and metadata file sRNA-seq: miRNA data**

Supp_File_5_40_mirna_counts_LF.xlsx

Supp_File_6_40_mirna_metadata_LF.csv

**Supplementary File S7 & S8- raw counts and metadata file sRNA-seq: piRNA data**

Suppl_File_7_piRNA_raw_counts.csv

Supp_File_8_pirna_metadata.csv

**Supplementary File S9 & S10- TEtranscripts RNA-seq raw counts output and metadata file**

Suppl_File_9_tetranscripts_raw_counts_Teonly.xlsx

Suppl_File_10_metadata_rnaseq_TEtranscripts.csv
